# “Now I feel like I can”: exploring e-commerce for self-care contraception in Kenya

**DOI:** 10.3389/fgwh.2026.1834359

**Published:** 2026-06-17

**Authors:** Pauline N. Wekesa, Emily Himes, Janelli Vallin, Julia Kramer, Serah Gitome, Sarah Okumu, Zachary A. Kwena, Lauren Suchman, Louisa Ndunyu, Beth Phillips, Elizabeth Bukusi, Jenny Liu

**Affiliations:** 1 Centre for Microbiology Research, Kenya Medical Research Institute, Nairobi, Kenya; 2 Department of Family and Community Medicine, University of California, San Francisco, San Francisco, CA, United States; 3 Institute for Health & Aging, University of California, San Francisco, San Francisco, CA, United States; 4 Department of Mechanical Engineering, University of Michigan, Ann Arbor, MI, United States; 5 Department of Public Health, Maseno University, Kisumu, Kenya

**Keywords:** contraception, e-commerce, Kenya, self-care, women

## Abstract

**Introduction:**

In Kenya, the growing e-commerce market presents new opportunities to expand access to self-care contraceptives. However, we know little about women's experiences and attitudes toward purchasing such products online.

**Methods:**

We used a two-phased approach to explore Kenyan women's perspectives on e-commerce and its potential as a platform for contraceptive access. In the formative phase, we conducted 61 in-depth interviews with a diverse sample of reproductive-aged women to assess general perceptions of e-commerce and attitudes toward using it for contraceptive purchases. We analyzed formative data through an iterative coding process followed by thematic analysis of relevant codes reports. In the second phase, using a human-centered design (HCD) approach, we observed a smaller group of Nairobi-based women of reproductive age (*n* = 8) as they navigated the website of a women's health-focused digital commerce platform to explore products, access information and make purchases. Post-website use interviews captured participants’ reflections on the experience. Research assistants compiled detailed notes from observations and interviews that the analysis team used to identify themes and build out analysis insights.

**Results:**

While some participants in the formative phase noted the convenience of online shopping, many were skeptical of e-commerce due to concerns about fraud or their own limited digital literacy and internet access. Similar issues with digital accessibility surfaced in the HCD phase; however, after a brief tutorial and direct experience using the website to browse and make purchases, HCD participants reported more positive perceptions of e-commerce. They emphasized the privacy, convenience, and autonomy that online purchasing offered, noting that these features were especially beneficial for accessing contraception.

**Discussion:**

Findings from the HCD phase highlight the potential for e-commerce to lessen the burden of external pressure from providers or family members on contraceptive decision-making and to help circumvent stigma-related barriers, especially for younger women. While HCD participants’ increased confidence in and comfort with e-commerce after exploring the digital commerce platform indicates promise for this self-care contraceptive access pathway, barriers to use identified by participants in both phases underscore the need for such digital interfaces to be implemented with in-person introductions that can build trust and offer technical guidance.

## Introduction

Contraceptive use in Kenya has increased from 32% of married women using a modern method in 2003 to 57% in 2022 (1) but 14% of women between the ages of 15–49 may have an unmet need for pregnancy prevention (1). Barriers to contraceptive access persist, including providers inhibiting user choice or refusing methods, often based on their own biases about method suitability (2, 3). Adolescent girls and unmarried women are particularly susceptible to judgment from providers or others, often citing lack of confidentiality and stigma as reasons not to seek contraception. Additionally, because younger women often receive information about contraception from their peers, hesitation to use contraception may stem from myths or misconceptions (4). Existing barriers to contraceptive access heighten women's preferences for more positive provider interactions, privacy, and convenience, which influence decisions about where they choose to obtain methods (5).

Given ongoing unmet contraceptive needs and access barriers, the World Health Organization supports sexual and reproductive health *self-care* as a promising approach in which women can use certain methods independently and engage with the formal health care system as needed (6). Such methods include daily contraceptive pills, emergency contraceptive pills, male and female condoms, and self-injectable contraception (6). Indeed, much of the Kenyan population already accesses self-care contraceptives outside of the public health system, for example, through private clinics or pharmacies (5, 7).

The growth of digital connectivity and e-commerce in low- and middle-income countries, including Kenya, presents an opportunity to further expand access to sexual and reproductive health self-care products. In 2014, only 14% of the Kenyan population used the internet (8). By early 2026, more than half the population was online with usage rates highest in urban areas (76%) and among people between the ages 15–34 (71%) (9). Similarly, e-commerce adoption has increased with consumers projected to reach 12.3 million in 2024 generating an expected revenue of 900 million USD (10). Though not a panacea for those who lack access to internet-enabled devices, access to information and self-care contraception through e-commerce could add a critical layer of autonomy for many women who struggle to obtain contraception through traditional health service channels (11). Notable consumer experiential challenges, however, have stymied the uptake of online shopping, including poor customer service, the inability to inquire about products in person, fear of being scammed and perceived security risks, and delivery delays, mistakes, and costs (12, 13). Such challenges may similarly impede the acceptability and feasibility of offering self-care contraceptives via e-commerce.

Our study aimed to explore the promise and limitations of offering self-care contraceptives via e-commerce in Kenya and the critical support elements consumers need to enable their informed choices about methods. As technology becomes increasingly prominent in health care across contexts, digital tools are playing a larger role in supporting sexual and reproductive health; a number of recent studies have examined their acceptability and impact in lower- and middle-income countries (LMICs) (14–17). However, studied tools often have a unidirectional flow of information, typically designed to disseminate content, provide education or offer digital reminders to improve adherence to medication regimens (14–17). Few studies have explored digital tools that provide information with an integrated e-commerce platform. Existing research in this area tends to rely on quantitative or survey–based data and often focuses on experienced e-commerce users (18–20).

In our qualitative study, we sought to address this gap in the literature by using a unique two-phased design that not only to captured women's general perceptions about e-commerce, but also provided insight into the lived experience of *using* e-commerce, including its benefits, challenges, and potential as an access point for self-care contraceptive methods. The range of qualitative methods used yielded depth and nuance that is lacking in the current literature. Our formative phase involved in-depth interviews (IDIs) with 61 women of reproductive age with varying levels of exposure to e-commerce. The second phase involved a human-centered design (HCD) research approach to observe women using the website of a digital commerce platform specializing in women's health and self-care needs, and later interviewing them to learn more about their experience. In both phases, we employed a thematic analysis approach to generate insights on how women of reproductive age perceive e-commerce, its challenges, benefits, and potential. Findings specific to obtaining contraceptives via e-commerce are valuable for understanding the acceptability of this modality as an access point for self-care contraceptives and can guide implementation strategies for digital health tools that support women seeking access to contraceptives or other self-care services through similar channels.

## Methods

### Study setting

The Innovations for Choice and Autonomy (ICAN) project, which began in late 2019, is a multi-country initiative aimed at deepening understanding of contraceptive decision-making and identifying implementation strategies for self-injection of subcutaneous depot medroxyprogesterone acetate (DMPA-SC) (21, 22). The Kenyan government approved DMPA-SC for contraceptive use in 2019, first allowing provider-administered DMPA-SC and later for self-injection by users themselves after an initial in-person training on the procedure with a trained provider (23). For this study, our aim was to explore how e-commerce could be used to facilitate access to self-care contraceptives, including self-injection of DMPA-SC, and improve autonomy of contraceptive decision-making. We conducted this study in Nairobi and Kisumu, Kenya, two geographically and culturally diverse cities. Kisumu lies on the shores of Lake Victoria in western Kenya and hosts a thriving fisherfolk community predominantly comprised of people from the Luo ethnic group. Nairobi is the capital city of Kenya and is home to people from all of Kenya's ethnic groups; more than 60% of its population lives in informal settlements where access to healthcare, including reproductive services, is limited (24). The contraceptive prevalence rate for both Kisumu and Nairobi counties is above 60% and the majority of women who use modern contraceptives prefer using injectable contraceptives, daily contraceptive pills, and condoms; with the introduction of DMPA-SC for self-injection, all of these method options fall into the category of self-care contraceptives (25).

### Study design

We collected data in two phases: the formative research phase and the HCD research phase. An aim of the formative research was to obtain a deeper understanding of the familiarity with and utilization of e-commerce websites among women, while we designed the HCD research and analysis phase to inform new solutions to address contraceptive needs through a digital commerce platform. Following the HCD research and analysis phases, we embarked on a co-design process with a community advisory board including idea generation, prototype development, and solution testing [details of the whole HCD process and the final solutions are reported elsewhere and not a focus of this manuscript (26)]. We partnered with a for-profit direct-to-consumer (DTC) business specializing in health and hygiene products, which began in Rwanda in 2016 and expanded to Kenya in 2019. The digital commerce platform offers customers multiple purchasing channels, including website product browsing, in-person purchasing with trained community agents, and remote ordering via phone with a call center, WhatsApp, or through an unstructured Supplementary Service Data (USSD) code. Customers are required to make payment immediately after completing their shopping so that the order can be confirmed and delivery initiated. Mobile payments are made using M-Pesa, a money transfer method via mobile phone. All purchases are fulfilled via at-home delivery and payment includes a delivery fee (27). We collected data through in-depth interviews (IDIs) in the formative research phase and both participant observations and IDIs with current and potential users of the website in the HCD research phase. Methods used for data collection and analysis for these activities are detailed in prior publications (21, 22), and also summarized as follows.

### Formative research phase

#### Recruitment and eligibility

Recruitment for the formative research phase began on February 16, 2021, and ended May 6, 2021, in both Kisumu and Nairobi Counties. We worked with community health promoters (CHPs) to identify potential participants through their community networks or through health facilities and local pharmacies offering contraceptive services in Nairobi and Kisumu. The study team conducted initial eligibility screening and purposively sampled women of reproductive age (15–45 years) to ensure diverse representation by age (15–19 years and 20–45 years), contraceptive use or non-use, marital status, and experience with DMPA-SC. We defined contraceptive users as those who were currently or previously using any known methods to prevent pregnancy, while non-users had never used any of these methods. We identified 61 participants (Kisumu *n* = 31, Nairobi *n* = 30) and interviewed them after they provided informed written consent. We received ethical approval to waive consent for minor participants due to the study's subject matter and the potential risks that involving parents or guardians could pose to participants.

#### Data collection

We conducted IDIs face-to-face using a semi-structured interview guide. Participants chose their preferred place that was convenient, quiet, and offered confidentiality. Trained research assistants (RAs) who were fluent in English, Swahili, and Dholuo conducted the interviews. Interviews lasted 1–2 h and were audio-recorded with the consent of the participant. Participants were reimbursed Ksh 1,000 (∼10 USD) for their time and travel. In addition to questions about participants' experiences with contraceptive decision-making, access, and use, interview guides included questions about using e-commerce, what support they needed to do so, reasons for choosing to shop via e-commerce vs. other sales channels, and views on using e-commerce to purchase contraceptives.

#### Data analysis

A team of researchers (PW, JV, LS, SG, ZK, LN) coded IDI transcripts through an iterative, rapid analysis process. The team completed two rounds of remote, synchronous collaborative coding to establish consistent coding application before reviewing the remaining transcripts independently. More details on the formative phase analysis are available in earlier publications (21, 22). For this paper, we generated code reports related to women's access to, experiences with, and interest in using e-commerce, and their perspectives on DTC purchasing of contraceptives or other self-care products. Three lead analysts (PW, JV, EH) reviewed the code reports employing a thematic analysis approach to draw insights from emerging themes (28). Findings were then discussed with the larger team to reach consensus.

### HCD research phase

#### Recruitment and eligibility

Recruitment for the HCD research phase began November 22, 2021, and ended March 3, 2022. Unlike the formative research phase, we conducted the HCD research phase exclusively in Nairobi County because the delivery systems and general operations of our partnered e-commerce business were more established in Nairobi than in other parts of Kenya at the time of our study. We worked with CHPs to identify participants who were 18–45 years, were using or were willing to use an e-commerce website, and were current or potential contraceptive users. We recruited eight participants for direct observations, of which seven completed interviews afterward (one participant was unable to find a suitable interview time).

#### Data collection

For participant observations, study participants received a Ksh 2,000 (∼20 USD) shopping voucher and, if they did not have their own device, an internet-enabled tablet to use for shopping on the website of the study's partnered digital commerce platform. To ensure that all the participants had basic familiarity with navigating the website, RAs gave a brief tutorial covering actions like using the search bar and drop-down menus and setting up an account if the participant did not have one already. We then asked participants to browse the website while also verbalizing what they were looking at (e.g., homepage, offerings including contraceptive methods) and thinking, and thereafter to purchase whatever they wanted using the gift card provided. Participants were observed by RAs to assess the ease of using the website and their ability to navigate and identify items that they wanted to purchase. Participants shared their thought processes as they made decisions about what to purchase and the challenges they encountered while navigating the website. RAs recorded their observations on an AEIOU observations framework template that captured participant experiences related to activities, environments, interactions, objects, and users (see Supplementary Appendix 1) (29). After two weeks, we contacted participants again to schedule an interview; in this two-week period, the ordered products were delivered, and therefore, participants were able to share feedback on the end-to-end consumer experience. During follow-up interviews, we asked participants about their experiences in seeking health services and products, contraceptive decision-making and use, and their comparative experience with using the e-commerce website during the observation. We also asked participants about their interaction with the digital commerce platform staff members, if relevant, and their satisfaction with the delivery process. Observations and interviews lasted 1–2 h each, and participants were reimbursed Ksh 1,000 (∼10 USD). Participants provided a written consent for both the observations and interviews in the language of their preference. We received ethical approval to waive consent for minor participants due to the study's subject matter and the potential risks that involving parents or guardians could pose to participants.

#### Data analysis

After conducting observations and interviews, RAs played back audio recordings and logged notes using post-observation and post-interview tools we developed (see Supplementary Appendix 1). RAs recorded participants' general experiences while shopping online, noting challenges faced and decision-making regarding purchases. For interviews, RAs recorded participants' views on seeking health services and products, family planning decision-making, and use, and experiences with e-commerce. Observation and interview notes were reviewed by a team of nine researchers (PW, SG, NC, JK, ZK, JL, EH, SO, JV) to identify significant themes raised by participants. The team met on a regular basis and using Miro, a digital whiteboard platform that supports collaborative analysis for remote teams, coalesced the emerging themes through affinity mapping. Themes were then refined into analysis insights, shared with all co-authors and finalized with their feedback. PW and EH then discussed findings from both phases, drawing connections between them, and shared a consolidated, written summary to the team for approval.

### Ethical approval

Ethical approval for both study protocols was obtained from the Kenya Medical Research Institute Scientific and Ethics Review Unit (KEMRI SERU #4013 and KEMRI SERU #4228) and the University of California, San Francisco Institutional Review Board (UCSF IRB #19-29805 and UCSF IRB #21-33997). Research permits were obtained from Kenya's National Commission for Science, Technology and Innovation (NACOSTI approval #464643 and NACOSTI approval #685972).

## Results

### Socio-demographic characteristics of participants

In the formative research phase, we interviewed 61 women of reproductive age from Nairobi and Kisumu (Table 1). Most were single (66%), aged 15–24 (59%), and reported a history of contraceptive use (59%). In the HCD research phase, eight women of reproductive age participated in the observed experience using the e-commerce website; all but one participated in the IDIs that followed. In this sample, all women were from urban Nairobi, 38% were single aged 18–24; 63% had used contraception before, and half had used the study's partnered digital commerce platform prior to enrolling in the study.

**Table 1:** Participant demographic characteristics.

Characteristics		Age 15–24	Age 25–45
Formative phase (*N* = 61)			
Marital status	Single	26	14
	Married	10	11
Contraceptive use	Never	18	7
	Prior or current use	18	18
Human-centered design research phase (*N* = 8)			
Marital status	Single	3	0
	Married	0	5
Contraceptive use	Never	1	2
	Prior or current use	2	3

We present our findings in two parts. First, we cover the results from our formative work on general perceptions of e-commerce and the potential of using it for contraceptive purchases. Participants described a mixed level of familiarity with e-commerce, a high degree of mistrust of the practice, and varying access to the internet. Second, we share findings from the experiences of the smaller group of women observed using the website of the study's partnered digital commerce platform, exploring, and sometimes purchasing, self-care products. For many HCD research participants, shopping for contraception online appealed to their desire for privacy, autonomy, and convenience when obtaining contraception; however, some practical challenges related to insufficient digital literacy, low awareness of e-commerce, and limited internet access surfaced. Table 2 summarizes our themes and key findings.

**Table 2:** Summary of themes and key findings.

Study phase	Themes	Key findings
Formative phase	Limited use of e-commerce	Many participants had never considered using e-commerce
	Reasons for non-use	Skepticism about e-commerce legitimacy and fear of scams were common
		Lack of access to appropriate devices limited use
	User vs. non-user perceptions	Those with experience cited convenience, better product availability, and pricing advantages
		Non-users were more likely to perceive e-commerce as expensive
	Limited access to internet-enabled devices	Many participants did not own an internet-enabled device
	Access through peers or family	Sharing or borrowing a device was possible for some, but compromised privacy for others
		Parents kept their own devices close, limiting access for adolescents
	E-commerce not viewed as a platform for contraceptive purchases	
	Lack of awareness	Low awareness of e-commerce as an access point for contraception among participants
	Trust and legitimacy constraints	Several participants stated preferences for in-person consultations
		Some participants shared heightened concerns about fraud
Human-centered design research phase	Hands-on engagement with e-commerce shifted perceptions	Engaging directly with website increased willingness to use e-commerce, including for contraception
	Platform usability and features	Participants reported positive experiences with browsing, pricing, delivery, and potential nurse interaction
		Reliable service features boosted trust in e-commerce
	Perceived benefits	Participants identified privacy and autonomy as key influences on interest in using e-commerce for contraceptive purchases
	E-commerce use revealed potential for improved care access	
	Prior contraceptive care experiences	Participants described stigma, pressure, and negative interactions when seeking contraceptive care previously
	Perceived e-commerce advantages	E-commerce seen as offering confidentiality, autonomy, and convenience
	Technical and digital literacy challenges	
	Usability barriers	Participants experienced difficulties with website navigation, search functions, and account setup
	Learning increased confidence	Support from research assistants improved confidence and perceived feasibility of use
	Access constraints	Limited data plans restricted browsing
		Participants without internet-enabled devices noted they could not access the website outside of the study context

### Women's general perspectives on e-commerce

#### Direct experience with e-commerce was limited; skepticism of its trustworthiness was prevalent

While many participants were aware of e-commerce, most had never used it to make purchases. A married 17-year-old expressed novelty for the idea, “I have never thought of buying anything on the internet.” For other participants, the lack of familiarity with the practice led to skepticism about its legitimacy. A single 19-year-old shared, “I have never been in a position to do that [shop online], maybe I feel that I can’t trust things being sold online or I am not well informed about online shopping.” Many participants feared being the victim of a scam. For example, some thought paying at the time of purchase “increased the chances of being conned.” Other participants worried they might not get value for their money or did not trust that they would receive the product they intended to order. A 38-year-old single participant explained that the lack of personal interaction in e-commerce was a barrier to her trusting the process and therefore using it: “…I am scared, there is a lot of fraud going on around here [e-commerce websites]. I would not be sure of what I am doing, not unless I have dealt with people directly, that is why I wouldn’t be tempted to go there.” Some participants speculated that not receiving the desired product could reflect compromised quality or even a safety issue. This was a concern for a single 33-year-old who also trusted the in-person sales more readily: “I am not confident because am not sure if the drug I’ll order is the real one or not. But when I find you in Kenyatta, it will be easy to direct me where I can get treated.” By referencing Kenyatta, a well-known hospital in Kenya, here the participant expressed her higher degree of confidence in physical settings over an e-commerce marketplace when purchasing medication. Overall, we found considerable hesitancy to engage with e-commerce and heightened concerns of being defrauded when women considered utilizing e-commerce for health care needs.

#### The benefits of e-commerce were appreciable, cost-savings were debatable

While this mistrust of e-commerce was common, participants also described certain benefits. Several women with e-commerce experience described positive experiences in purchasing clothes, electronics, or baby products, for example. The convenience of buying online from the comfort of their home was highlighted by several participants, including an unmarried 30-year-old:

“The advantages of online is if you buy something online it will be delivered at your doorstep so you don't go after the item, but with the shops I have to walk from shop to shop looking for the item maybe sometimes I don't have the time to go to the shops so at least they deliver at your doorstep.”

Several participants mentioned that paying on delivery was preferred and they were pleased when that option was available because it minimized the risk of fraud. A single 25-year-old participant shared her positive experience with both delivery and payment procedures: “They were fast and the price that you agree on, you pay after delivery, on delivery so they cannot even steal from you because they send honest people I think, from the company.” Some participants reported making purchases through e-commerce because the product they sought simply was not available in stores. Other participants were enticed by attractive discounts on items they deemed to be of high quality. Participants readily shared that the ability to compare prices and browse for discounts was appealing; few formative research participants organically mentioned seeking more general product information though some HCD research participants discussed using the internet to learn about contraception (see further below). A single 17-year-old reported making an online purchase when she found a good deal on shoes; she shared, “The advantage is that some things are very cheap compared to the stores plus you are not paying for the transport so while you order it you are sure that it will come to you safe and they will bring the exact thing.” While cost-savings were a motivating factor among participants who had used e-commerce, among participants who had no prior experience, many perceived e-commerce to be *more* expensive and/or assumed the process would require extra fees, especially for delivery, on top of the cost of the product. One such participant, a single 16-year-old, generalized, “But with the apps, they sell things at high prices.” Participants with prior e-commerce experience were in the minority, but they were most likely to highlight its benefits. This suggests either positive baseline attitudes towards e-commerce drive adoption or that exposure to it fosters greater trust and acceptance.

#### Access to internet-enabled devices was limited; sharing devices was only acceptable to some

One reason for infrequent e-commerce use cited by participants was not having an appropriate device for connecting to the internet. A single 42-year-old participant explained that she had never used e-commerce because she did not own a smartphone and was unfamiliar with their capabilities. “You know we have the small phones which don’t have Facebook! … I only know that phones are for calling and receiving calls. That's what I have and what I have always had.” When participants had no access to the right device or technology, they were unlikely to conceive of e-commerce as a way to purchase goods. However, most participants who did not own a phone or an internet-enabled device knew someone who did, but the acceptability of borrowing a device to make online purchases varied. Some participants were confident that a sibling or a friend would lend their phone; another participant stated that the e-commerce purchase she made was executed by her husband on his device. However, issues with device sharing were raised. A 42-year-old unmarried participant expressed concerns about the confidentiality of using someone else's device, “It won’t be easy because someone will ask you what you need the phone for. There will be a lot of questions.” A single 16-year-old stated that even though both her parents owned devices, using them was not an option because her parents always kept them close, for example in a pocket. Overall, limited access to e-commerce was common for a variety of reasons—participants rarely owned computers, some mentioned limited data plans or owning phones that were not internet-enabled, and privacy concerns reduced their willingness to share devices for online purchases.

#### E-commerce was not typically viewed as a platform for contraceptive purchases

When asked about knowledge of applications or websites where one could purchase contraception, many participants responded with confusion or made clear their lack of awareness about this option. A married 32-year-old contraceptive user responded, “Honestly I do not understand now, I am just in the dark,” when asked if she knew how to buy personal care products or contraception online. Another participant, aged 33 years and single, queried, “Is it [contraception] available online? I have never seen [that].” The idea of buying contraceptive products through e-commerce amplified some participants' desire for in-person interactions when making purchases. Several participants expressed greater comfort meeting with a health care provider face-to-face when seeking contraception, allowing them to ask questions and ensure a method was safe. A 20-year-old single contraceptive user explained, “I prefer going to the hospital so that I get the guidance and counseling, then it is you to choose which type of family planning to use.” For participants who harbored fears about e-commerce fraud, the idea of purchasing contraception online heightened the perceived risks. Another single 17-year-old contraceptive user acknowledged, “I don’t trust them with that sector because maybe something has expired… They remove that box with the expiry date and put another box and now they deliver.” One participant mentioned the privacy of having a method delivered to your home, and another liked the convenience of not traveling to and waiting at the pharmacist. However, most participants were apprehensive or unsure about purchasing contraception through e-commerce; the idea seemed to prompt concerns and confusion more than interest.

### Women's perspectives on engaging with an e-commerce website

#### Positive experiences with the e-commerce website boosted trust and interest in the process

Having the opportunity to use e-commerce, especially after some basic instructions from the RAs, yielded positive experiences among HCD research participants. Participants were pleased with the level of customer service, the delivery and payment protocols, and the quality and accuracy of the products they received. As this 33-year-old married contraceptive user said, “The minute you place an order, they call you to confirm. If a product is not available, they ask if you want an alternative or if you want to wait. I think they are doing a good job.” Similarly, delivery drivers kept participants informed about when and where orders would arrive. Further, women appreciated having a health-specific contact in the form of a nurse for questions about contraceptive options. As this married 33-year-old contraceptive user explained, “If one wanted more information, [the website] provided alternative channels, like a phone number and an inquiry form, or the option to book a consultation with a nurse to discuss DMPA-SC.” This human touch point was reassuring for some participants, easing anxieties about navigating the contraceptive information independently.

During observations, a few participants purchased contraceptives, such as condoms and daily contraceptive pills. These participants, and others, noted how browsing for and buying contraceptives through this e-commerce website offered privacy and autonomy benefits. A participant who purchased daily contraceptive pills during the observation explained that she typically used a “coded language” when she went to the pharmacist for contraception to prevent men from understanding what she was purchasing. Using e-commerce helped her to realize that this way of accessing contraception could be preferable because the products arrived “well wrapped,” ensuring no one would know what it was. A 35-year-old married contraceptive user echoed this sentiment about e-commerce privacy and the advantage of avoiding judgment in brick-and-mortar stores:

“Purchasing the condoms or other contraceptives is much more comfortable than from the physical shops where people look at others badly and make them feel uncomfortable. When you purchase the contraceptives at [this website], they are well packaged in a white paper bag. No one can tell what they are. Again, when you order it is only you and your gadget that will know you are placing an order for the same. No one else will know. [This website] is a better option for me.”

Furthermore, a single 24-year-old contraceptive user shared that the e-commerce website felt like a “safe space” for learning about contraceptive options in her own time. She expressed concern about rushing the decision of what method to use because many of her peers had struggled with side effects. Engaging with the website through the study shifted some participants' perspective, making them more open to the advantages of online access to contraception.

Finally, most participants responded favorably to the prices of the products available, including delivery fees. Some were aware of prices on other websites or in-person shops and could say that the prices were fair or even better. A single 24-year-old who had used the digital commerce platform prior to the study shared, “This women's health website is a bit cheaper, especially when they have discounts. And then you know you can be given deliveries depending on the time you want. If you want it now, tomorrow, it is cheaper and at your doorstep.” Though most participants commented that the prices were fair, one participant noted the condoms were expensive and, therefore, opted not to purchase them during the observation.

#### Reflections on the privacy, autonomy and convenience of accessing contraception through e-commerce

Even when they did not purchase a contraceptive method during the observation, many participants recognized that buying contraception online was a confidential, convenient, and self-directed option. This realization often came after using the website and reflecting on their own experiences accessing contraception in the past. Several participants were already utilizing the internet to learn about contraception; Google or other sites provided the information they sought in a private and unpressured way. A single, 24-year-old contraceptive user, whose boyfriend and sister disapproved of contraception, explained why using the internet to access contraceptive information was the best option for her:

“I learned about family planning methods through Google, actually. It's safer and more secure to look it up on your own time, and it is quite private. So if I want to google effects of implants and IUDs [intrauterine devices], I don't have to ask my friend and not being sure how they will react to the question, so I prefer Google.”

In contrast to the ease of independently seeking information online, several participants reflected on unpleasant contraceptive counseling sessions they had experienced in clinics and hospitals. One participant shared that a male provider had flirted with her, making her uncomfortable and unable to focus on her decision about contraception. Another felt the provider was pushy, and she did not have an opportunity to voice her opinions about what method to use. A 35-year-old married user of contraception stated that her questions to a nurse were met with frustration:

“I was asking many questions about the method, whether it is safe, if I would get pregnant while using it. I kept asking the nurse whether it was the right method, whether I should do pills, injection, etc. The nurse got mad at me and told me, if I do not want [a method], and I have not decided, that I should leave and come back another day.”

Later, this same participant reflected positively on the prospect of buying contraceptives through e-commerce, “[in the] privacy in my home, I can always do that. Plus, I don’t get an attitude [from a provider].” As they used e-commerce through the study, participants connected that online access offered a way to circumvent prior undesirable contraceptive-seeking experiences.

Several participants highlighted that shopping in person for contraception made one susceptible to judgment from others. They noted that the stigma surrounding contraceptive use was particularly problematic for younger women, who often feared seeking contraception through a provider or might know local shop owners in the community. During the observation, a married 26-year-old who did not use contraception described how e-commerce could benefit younger women who faced barriers to obtaining contraception:

“It is a good idea for the young women of the age around 20 years to buy their contraception from the online shop like [this website]. This is because sometimes they go to the health facility, and they fear talking about family planning, yet they are giving birth without planning. We should encourage them to use family planning because they might fear to go to the pharmacy, or to the health facility to get the methods, but when they order online, it will just be brought to them where they are. This will be very good for the youth.”

This layer or privacy and confidentiality was another potential draw of e-commerce as participants reflected on common barriers to contraception and their experience using the website.

Finally, participants identified convenience as a benefit of shopping online for contraception because most were accustomed to traveling and waiting in a queue when seeking such services in person. A married 35-year-old participant who used contraception, proposed that the convenience of shopping for contraception online could be compounded by bundling her purchases together and buying a range of products at the same time, something that was possible on our partnered digital commerce platform.

#### Insufficient digital literacy and internet access limited the feasibility and accessibility of e-commerce

During observations, many participants—especially those without prior experience shopping online— had mixed experiences navigating the website; we noted difficulties related to both general and digital literacy. While participants were instructed on how to use the search bar to find products of interest, some struggled with spelling the items correctly and failed to find what they were looking for without assistance. Some participants found the site navigation challenging: a single 24-year-old who had not used the website before informally gave the site a rating of “6 out 10” for usability, and another married 35-year-old participant found the many menu bar subheadings confusing, even though she had prior experience using the website. Despite these challenges, participants still endorsed the potential of e-commerce. The married 26-year-old participant who struggled to use the search bar said, “I thought that browsing on the website was difficult, but when you [the RA] showed me how to do it, I now think I can do my shopping on the website on my own.” ​A married 35-year-old who had not used the website before believed women would benefit from support and education, which could lead to greater adoption of e-commerce. She explained, “Women need education to be able to shop online…” Reflecting on her own experience, she shared, “In my mind, I wasn’t even thinking of ever shopping online, I was just comfortable doing whatever I do daily, but now I feel like I can shop online.” For most of the participants that faced challenges navigating the website, learning how to use it effectively boosted their confidence and, at times, prompted ideas about how other women could be supported and encouraged to engage with e-commerce.

A few participants faced technical difficulties during the observation. One participant could not recall her email address, which she rarely used, and therefore had trouble completing her purchase. Connectivity was occasionally an issue, but limited data for browsing the website was more common. Several times during observations, participants reached their data limit while exploring the website, which they found frustrating. A single 24-year-old user of the website highlighted internet access issues in her interview: “Women would be discouraged from using [this website] if they don’t have [data] bundles. Obviously, you have to have a smartphone…” Another participant, 33 years old, married, and a prior user of the website, acknowledged that, while a lot of women have phones, many do not own smartphones. Therefore, accessibility through varied formats would be beneficial to potential customers. One option, as she explained, was USSD: “Women should be given the option to order through the USSD since there are some who don’t have smartphone but would love to purchase.” Unbeknownst to most participants at the time, the digital commerce platform was already implementing USSD as a payment method. Another solution for limited internet access involved using a “sales agent”—an individual contracted by the business to place orders on behalf of customers using their own device, in exchange for a small commission; at least one participant reported using this means to make purchases.

## Discussion

Our two-phased study design complemented perceptions of e-commerce from formative interviews with a deeper understanding of implementation barriers and potential promises of purchasing contraceptives online by observing direct engagement with our study's partnered digital commerce platform. Participants in our diverse formative research sample expressed varied levels of familiarity with e-commerce, from indifference or wariness, citing concerns about scams and online fraud among those naïve to e-commerce, to acknowledging its convenience among those with prior e-commerce experience. Concerns about general e-commerce use were heightened when it came to purchasing contraception online; some feared counterfeit products and others preferred face-to-face interactions with providers. For those without phones, many noted that borrowing one to make a contraceptive purchase would compromise their privacy. When they interacted with the e-commerce website, however, HCD research participants expressed positive experiences with customer service, found costs inclusive of delivery to be reasonable, and felt it was a secure and trustworthy way to purchase goods. Reflecting on their own previous in-person experiences with accessing contraceptives, many participants recognized the potential of e-commerce to reduce the stigma and inconvenience of procuring methods at clinics or pharmacies. For these participants, the idea of using the internet to educate themselves about contraceptive options and execute on a decision in their own time and on their own terms was appealing.

While our results suggest there is potential for e-commerce to increase self-care contraceptive access, the challenges encountered by our participants offer insights into ways to make the channel more accessible for facilitating contraceptive self-care. There were several participants who reported not owning a phone, and among these, many were wary of the consequences of borrowing a device for e-commerce use. This aligns with research highlighting the privacy risks associated with shared device usage, particularly common in rural areas where phone ownership rates are lower (30). While the United Nations promotes increased phone ownership as a means of empowering women under Sustainable Development Goal 5 (gender equality) (31), we observed that simply owning a phone did not guarantee access to the internet. The type of device, the data plan, and the reliability of the internet connection affected the feasibility of using e-commerce. Addressing structural aspects of the digital divide like digital infrastructure and smartphone ownership is beyond the scope of this paper, though improvements are ongoing and may eventually render some of these issues obsolete (32–34), For now, our findings underscore the need for digital interfaces to be designed with technical limitations in mind. Indeed, our partnered digital commerce platform incorporated such constraints into their operational model by offering multiple ways to make purchases—through sales agents who placed orders on behalf of customers, a call center, USSD, WhatsApp, and an on-call nurse for confidential consultations—which nearly all study participants were unaware of prior to being in the study. As new tools are developed, testing of digital health interfaces should include diverse user groups and account for the different ways people may access them (35). It is especially important to assess alternative access modalities in contexts where smartphones and broadband are not the norm. As digital solutions expand to improve health care access and quality, overlooking this need could exacerbate existing health inequities (36).

Digital literacy also emerged as an accessibility challenge with several women requiring coaching to navigate the website. The government of Kenya has developed many school-based initiatives to advance digital skills, which may be especially impactful for girls, but such offerings for most women of reproductive age are lacking (32, 37). Further, the gender digital divide is about more than a lack of education; social norms in many places discourage women's use of the internet, undermining their confidence and limiting adoption of digital tools (38, 39). In the same vein, our formative work indicated that e-commerce was often not perceived as an option, especially for contraceptive purchases. However, our HCD work showed that women were receptive to digital skills education and their self-efficacy in using the digital platform increased with minimal support. As our HCD co-design process evolved within the ICAN project, we recognized the value of human connection as a component of the digital interface and this was integrated into our final solution (26). In settings where there is hesitancy to engage with digital health tools, a “warm handoff” could be useful for increasing adoption, where interpersonal engagement could boost interest, provide technical guidance, and reassure users about fraud concerns. Community health workers or peer educators, with their established relationships in the community, are uniquely positioned to introduce women to the idea of e-commerce for self-care contraceptives and could support their initial use of such platforms (36).

Addressing digital access barriers and designing interfaces that accommodate varying levels of experience and connectivity can help increase adoption of digital health tools among women, including in the realm of reproductive health. Our findings suggest that, with the right support and greater awareness of online contraceptive services, women would use these platforms because of the identified benefits of confidentiality, autonomy, and convenience. Having the means to browse contraceptive methods, compare options, and place orders without traveling to a clinic may be especially valuable in contexts where stigma, privacy concerns, or geographic distance limit access to care.

### Limitations

A strength of our study was use of a two-phased design to contrast general perceptions of e-commerce with participants' direct experiences interacting with the website of our partnered digital commerce platform. Differences in our sample selection parameters in each phase may limit the generalizability of our findings, however, we felt the benefits of generating complementary data offered deeper learnings than each singular activity alone and outweighed potential limitations. Future studies could follow women unfamiliar with e-commerce through a digital skills intervention to better understand the impact of increased awareness and education on patterns of e-commerce use. Additionally, our research focused on a single e-commerce website, restricting our ability to generalize findings to other platforms, and inclusion of a gift card may have introduced bias by encouraging participants to complete purchases despite usability challenges. However, it also allowed for a comprehensive evaluation of the full e-commerce experience.

## Conclusion

Our results yielded three key takeaways regarding Kenyan women's use of e-commerce and its potential to increase access to contraception. First, we found mixed perceptions of e-commerce and considerable mistrust of the practice. Second, challenges related to digital literacy and limited access to data plans and devices emerged, limiting the accessibility of DTC e-commerce shopping. Finally, more favorable attitudes about e-commerce and interest in using it to purchase contraception among potential users emerged after direct experience utilizing an e-commerce website. This shift suggests that exposure to and support with e-commerce may help adoption, thus opening up new access points for self-care contraception. To fully realize this potential, design, testing, and implementation of new digital health interfaces should account for a spectrum of digital readiness and experience.

## Data Availability

The datasets presented in this article are not readily available because data described in the manuscript will be made available upon request, with written approval for the proposed analysis from the Kenya Medical Research Institute (KEMRI) Scientific and Ethics Review Unit (SERU). Their application forms and guidelines can be accessed at https://www.kemri.org/seru. To request these data, please contact either the authors or the KEMRI SERU at seru@kemri.org. Data cannot be shared publicly because this study was conducted with approval from the KEMRI SERU, which requires that we release data from Kenyan studies (including de-identified data) only after they have provided their written approval for additional analyses. Requests to access the datasets should be directed to seru@kemri.org or beth.phillips@ucsf.edu.
