# Supplementary material for: “Now I feel like I can”: exploring e-commerce for self-care contraception in Kenya
Source: Front Glob Womens Health. 2026 Jun 17;7:1834359. doi: 10.3389/fgwh.2026.1834359 (PMC13318939; doi:10.3389/fgwh.2026.1834359)
Supplement: Supplementary file 1 [file Datasheet1.docx]

1. Kasha User Observation Note-Taking Template

Observation date and time: _____________________________ Participant ID: _______________________________

Location: __________________________________________ Observer: _________________________________

# **Detailed field notes:** Please listen back to your observation and fill in each section with detailed notes including the participant’s likes and dislikes and positive and negative emotions.

| **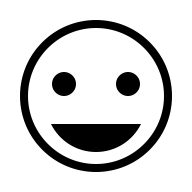Level of satisfaction with the Kasha website** | **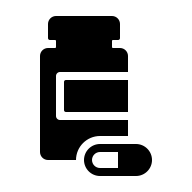What did they think of the offerings?** | **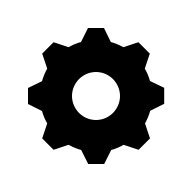What changes would they make to the website?** | **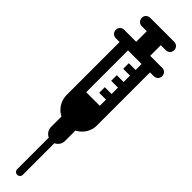Purchased contraception elsewhere** | **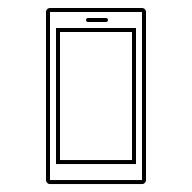Would they continue to use Kasha?** |
| --- | --- | --- | --- | --- |
|  |  |  |  |  |

# **Additional information from the participant:** (Please add any notes that do not fit into the categories above. You do NOT need to write the probes and responses word for word, just a summary is okay).

# **Key Take-aways:** Please answer the questions below.

1. What are the interviewee’s key needs?
2. What are their pain points/frustrations?

1. Think about developing a persona for your interviewee. How did their individual background influence their behavior?

**Name:**

**Occupation:**

**Location:**

**Goals and Needs:**

**Pain points:**

**Relevant behavior patterns:**

**Personality:**

1. Think about creating an ecosystem map for your interviewee. What factors (individual, interpersonal, organizational, community, and public policy) influence the participants user journey or job the most?

**Individual (knowledge, attitudes, skills):**

**Interpersonal (family, friends, social networks):**

**Organizational (organizations, schools, workplaces):**

**Community (access, connectedness, spaces):**

**Public policy (national, local laws and policy):**

1. What type of behavioral biases did you observe?

**Research Assistant Observation Guide for Observing Kasha Users and Potential Users: Key Questions for Note-Taking**

You will observe what goes on in the participant’s experience shopping on Kasha using the form attached to this guide.

[DO NOT ASK THESE QUESTIONS, TRY TO UNDERSTAND THROUGH PASSIVE OBSERVATION. IF THE PERSON DISCUSSES THEIR USE OF REPRODUCTIVE SERVICES TO YOU YOU CAN ASK CLARIFYING QUESTIONS]

**Situation:** Give a brief description of what is happening overall. What was the main purpose?

**Interpretation:** Given what you observed, what did you think about the effect of what happened on the participant? How would you describe the overall nature of the interaction?

**Time spent:** Note the approximate time the situation began and the approximate time the situation ended.

**Activities:** Note the specific actions and processes observed

**Environments:** Note the context of the place where activities are observed

**Interactions:** Note the interactions between different people, and the interactions between people and artifacts (i.e., between the participant and the website features).

**Objects:** Note the items within the environment and all the ways they are used (i.e., items and features of the website)

**Users:** The observable behaviors, relationships, and needs of the participant being observed

**Subject ID _______________________________ Date ____________________ Observer initials ____________**

| **Detailed description of the situation/event:** | | | **Interpretation of the situation / event:** | | |
| --- | --- | --- | --- | --- | --- |
| **Start time of situation/event:** | | | **End time of situation/event:** | | |
| **Activities**  Actions and processes observed | **Environments**  Context of the place where activities are observed | **Interactions**  Interactions between different people, and between people and artifacts (i.e., between the participant and the website features) | | **Objects**  Items within the environment and all the ways they are used (i.e., items and features of the website) | **Users**  The observable behaviors, relationships, and needs of the participant being observed |
|  |  |  | |  |  |

1. Kasha User Interview Note-Taking Template

Interview date and time: _____________________________ Participant ID: _______________________________

Location: __________________________________________ Interviewer: _________________________________

# **Detailed field notes:** Please listen back your interview and fill in each section with detailed notes including the participant’s likes and dislikes and positive and negative emotions.

| **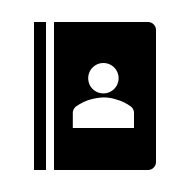Personal Background** | **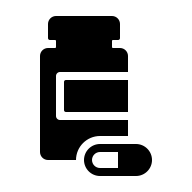Seeking Health Services or Products** | **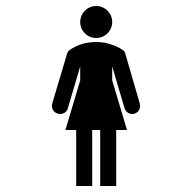Family Planning Decision Making and Use** | **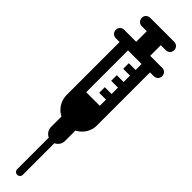Experiences and perspectives on Self-Injection** | **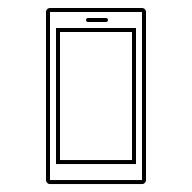Experiences with e-commerce** |
| --- | --- | --- | --- | --- |
|  |  |  |  |  |

# **Additional information from the participant:** (Please add any notes that do not fit into the categories above. You do NOT need to write the probes and responses word for word, just a summary is okay).

# **Key Take-aways:** Please answer the questions below.

1. What are the interviewee’s key needs?
2. What are their pain points/frustrations?

1. Think about developing a persona for your interviewee. How did their individual background influence their behavior?

**Name:**

**Occupation:**

**Location:**

**Goals and Needs:**

**Pain points:**

**Relevant behavior patterns:**

**Personality:**

1. Think about creating an ecosystem map for your interviewee. What factors (individual, interpersonal, organizational, community, and public policy) influence the participants user journey or job the most?

**Individual (knowledge, attitudes, skills):**

**Interpersonal (family, friends, social networks):**

**Organizational (organizations, schools, workplaces):**

**Community (access, connectedness, spaces):**

**Public policy (national, local laws and policy):**

1. What type of behavioral biases did you observe?
